# Gastroprotective effects of chlorella derived lutein via anti-inflammatory antioxidant and acid enzyme modulation

**DOI:** 10.1016/j.isci.2026.117661

**Published:** 2026-10-06

**Authors:** Insaf Bahrini, Maha Alazami, Muna Alamoudi

**Affiliations:** 1 Department of Biology, College of Science, University of Ha’il, Hail, Saudi Arabia

**Keywords:** ulcer, lutein, inflammation, antioxidant, algae, pigment

## Abstract

Induction of gastric ulcer by ethanol is associated with inflammation, oxidative stress, and impaired gastric mucosal defense. The current work was performed to determine the effect of lutein from Chlorella on ethanol-induced gastric ulcers in rats. Lutein caused significant decrease in gastric ulcer index and ulcer area (by 80.8%) and had beneficial effects on hematological and inflammatory status as seen from the 38% reduction in neutrophil, 57% reduction in myeloperoxidase activity, and 83% reduction in neutrophilic infiltration. At a dose of 200 mg/kg, lutein significantly lowered cyclooxygenase activity (50%), C-reactive protein (67%), prostaglandin E2 (55%), and nitric oxide (39%) levels. Lutein caused decrease in oxidative stress evidenced from 75% to 69% reduction in TBARS and hydrogen peroxide, and increase in the activities of superoxide dismutase, catalase, and glutathione peroxidase. Lutein exhibited dose-dependent inhibition of gastric H^+^K^+^ATPase activity and preservation of gastric mucosal integrity.

## Highlights

• Lutein protects against ethanol-induced gastric ulcers in rats
• Lutein reduces neutrophil infiltration and improves the lymphocyte/neutrophil balance
• Lutein decreases MPO, COX, CRP, and PGE_2_, reducing gastric inflammation
• Lutein enhances antioxidant defense and reduces ulcer area and ulcer index

## Graphical abstract

**Figure:**
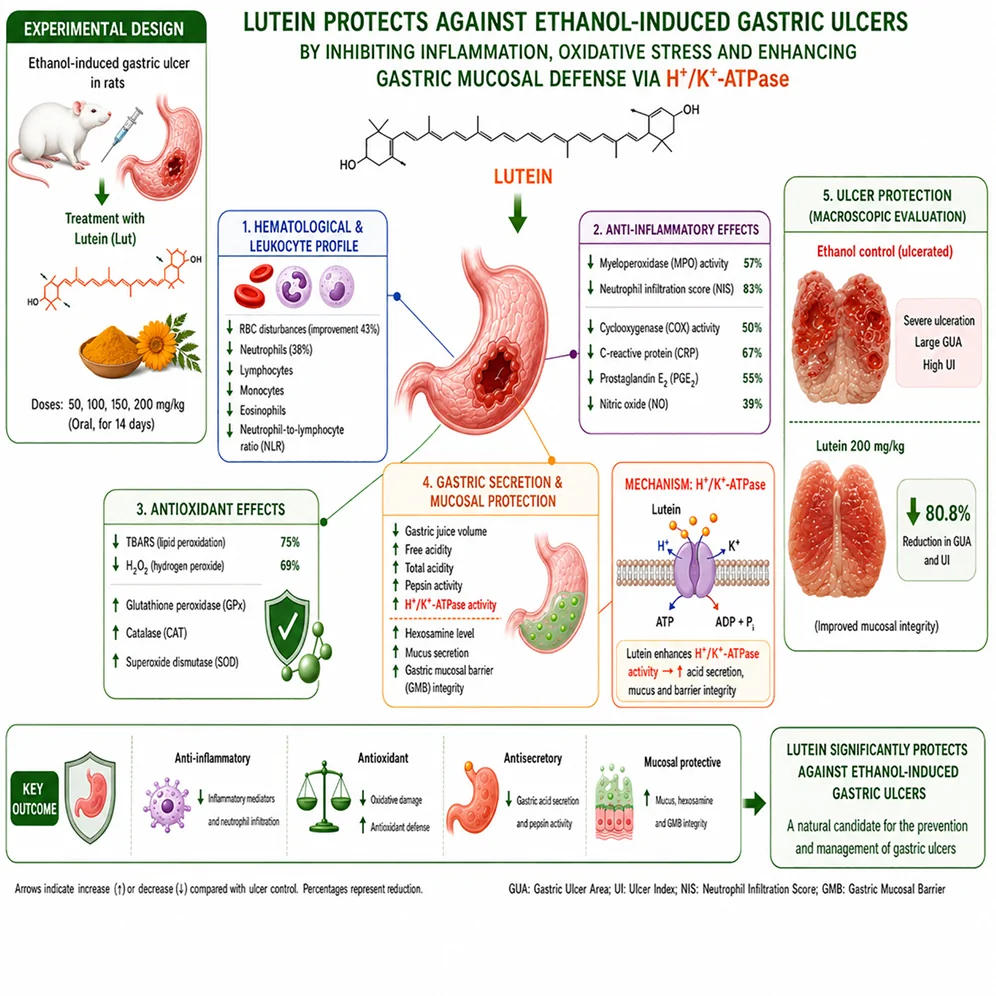

## Teaser

Biological sciences; Medical biochemistry; Natural product biochemistry

## Introduction

Gastric ulceration is a common gastrointestinal disorder, affecting approximately 8%–10% of the global population, with around 5% of cases developing gastric ulcers. This condition has multiple causes, including stress, alcohol consumption, smoking, *Helicobacter pylori* infection, synthetic food additives, and other medications that increase gastric acid and pepsin secretion.^1^ Currently, synthetic food additives, particularly colorants, are widely used in the food industry, but they may have harmful long-term effects on consumer health. For instance, tartrazine has been linked to allergic reactions and hypersensitivity, while quinoline yellow and azorubine are associated with genotoxicity, hyperactivity, and adrenal tumors. Allura red can cause immune system tumors, hyperactivity, and allergy-like reactions, along with toxic effects on various organs, including gastric, hepatic, renal, and reproductive functions.^2^ Due to these adverse effects, the food industry is increasingly shifting toward natural colorants in food, pharmaceuticals, and cosmetics. Natural colorants provide appealing colors for consumers. They also offer multiple health benefits, including antioxidant activity, protection against pathogenic microorganisms, and support for the function and health of various organs.^3^^,^^4^^,^^5^^,^^6^^,^^7^ Many conventional treatments for gastric ulcers can cause serious side effects. Patients often develop gynecomastia, hypoacidity, impotence, osteoporotic fractures, hypergastrinemia, or an increased risk of cardiovascular problems.^8^^,^^9^ Given the adverse effects of conventional treatments for gastric ulcers and the associated risks of gastric and intestinal disturbances, the use of natural compounds offers numerous advantages. These compounds are non-toxic, can improve gastric disorders without side effects, and provide a wide range of health benefits. These include the prevention of inflammation, enhancement of antioxidant defenses, modulation of immune responses, and various other beneficial effects.^10^^,^^11^^,^^12^^,^^13^^,^^14^

Microalgae are an important source of natural pigments and bioactive metabolites with numerous health benefits. Their rapid growth and low resource requirements make them suitable for sustainable production in a variety of environments. Rich in carotenoids, phycobiliproteins, and chlorophylls, these pigments exhibit potent antioxidant, anti-inflammatory, and antimicrobial properties, helping protect cells from oxidative stress.^15^^,^^16^ Microalgae have wide applications in the pharmaceutical, cosmetic, and food industries, providing an eco-friendly alternative to synthetic dyes. Among them, Chlorella stands out as a particularly sustainable and environmentally friendly source of bioactive compounds. It is rich in proteins, essential fatty acids, polysaccharides, vitamins, and natural pigments, offering antioxidants, anti-inflammatory, and immune-supporting effects. Its applications span the pharmaceutical, cosmetic, food, and nutraceutical industries, contributing to the prevention of metabolic disorders and supporting overall immune health.^17^^,^^18^^,^^19^^,^^20^ Due to its low environmental impact and rapid cultivation, Chlorella plays a key role in promoting sustainable solutions for human health and ecosystem preservation. Among algal-derived pigments, Lut is a prominent xanthophyll carotenoid (C_40_H_56_O_2_) commonly found in eggs as well as in dark green and yellow vegetables and fruits. Lut is highly valued for eye health as a functional food because it accumulates in the macula lutea, where it helps protect the retina from oxidative damage. In addition, Lut exhibits a broad range of pharmacological activities, including anti-inflammatory, antidiabetic, antioxidant, anti-apoptotic, and anticancer effects.^21^^,^^22^^,^^23^^,^^24^

No research, thus far, has ever examined the influence of Lut on the infiltration of lymphocytes and its subsequent inflammation in the case of ulcers in the gastric mucosa. In this case, our study marks the first instance of evaluating the influence of Lut on several immunological factors, such as the number of neutrophils and lymphocytes, NLR, and neutrophil infiltration score. Additionally, we explored the anti-inflammatory property of Lut by assessing the level of activity of some enzymes, as MPO and PGE2, responsible for inflammation in gastric mucosa. The level of oxidative stress induced by neutrophils was also considered.

## Results

### Purity assessment of isolated Lut

Our results demonstrate that the isolation protocol employed to extract Lut is effective in obtaining high-quality pigment. The purity of the isolated Lut was assessed by spectrophotometric measurement at 445 nm, yielding a purity level of 88% compared to a commercial Lut standard. This high degree of purity indicates that the extraction and purification method successfully minimizes impurities, ensuring the reliability of the Lut for subsequent applications, including research and pharmaceutical formulation.

### Lut absorbance and FTIR analysis

The analysis of the absorbance curve for the isolated Lut reveals the presence of two distinct peaks: a major peak at 445 nm, which corresponds to the characteristic absorption of Lut, indicating the presence of pure Lut or its primary isomers, and a secondary peak at 475 nm, representing additional absorption by Lut or its derivatives. This study confirms the effectiveness of the Lut extraction process, demonstrating both the presence and purity of the extracted pigment (Figure 1A).

**Figure 1:**
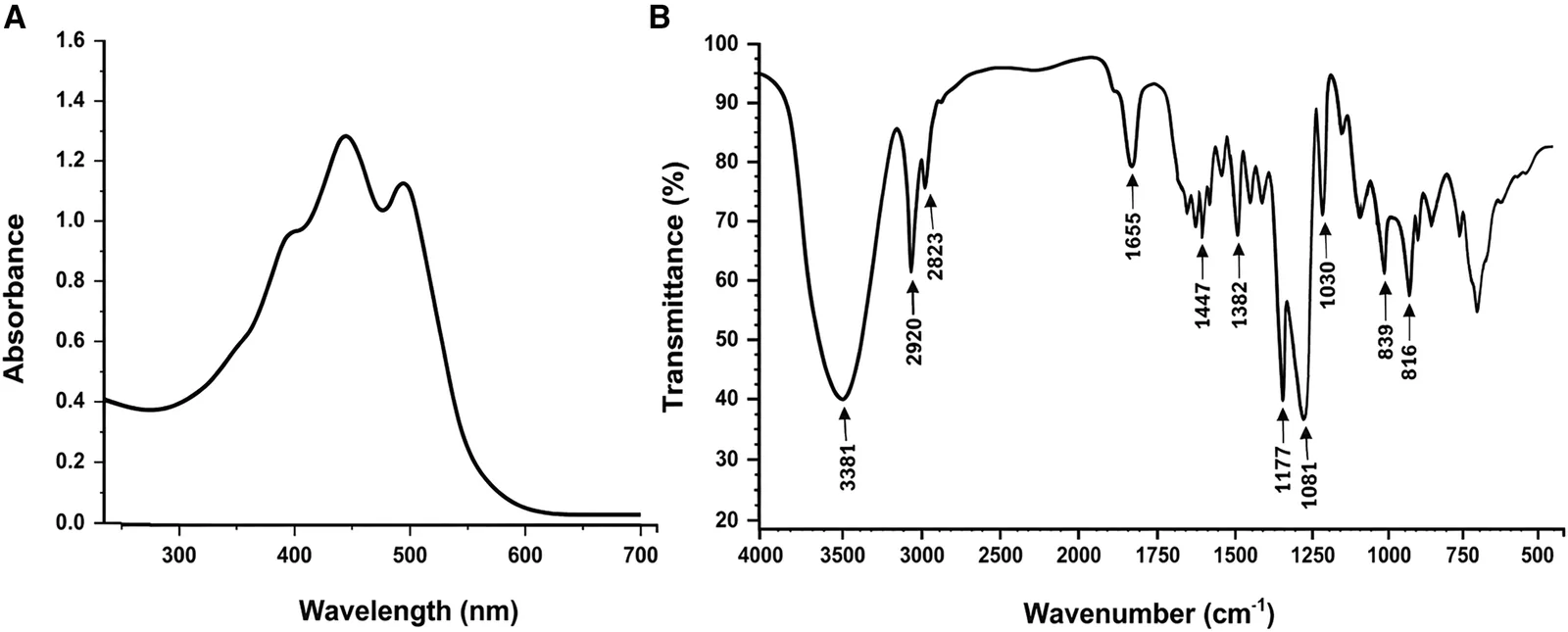
Characterization of lutein isolated from Chlorella algae (A) UV-Vis absorbance spectrum of isolated lutein. (B) FTIR spectrum of isolated lutein confirming its characteristic functional groups.

The FT-IR spectral analysis of Lut revealed a broad absorption band at 3,381 cm^−1^, corresponding to O–H stretching vibrations. Peaks at 2,920 and 2,823 cm^−1^ were attributed to C–H stretching. A distinct peak at 1,655 cm^−1^ indicated the presence of C=C stretching, characteristic of alkenes. The peak at 1,443 cm^−1^ corresponded to C–H bending. Additional peaks observed at 1,362, 1,177, 1,081, and 1,030 cm^−1^ were indicative of C–O stretching in alcohols. Peaks at 839 and 816 cm^−1^ were associated with C–H out-of-plane bending in aromatic rings (Figure 1B).

### Effect of Lut on gastric mucosal leukocytes in ulcerated rats

Our results, presented in Figure 2, show that gastric ulcers lead to an increase in the total number of leukocytes (A). Qualitatively, the study demonstrates that gastric ulcers cause a significant and marked increase in the number of neutrophils (B) and eosinophils (C), along with a reduction in lymphocytes (D) and monocytes (E). In contrast, in ulcerated rats treated with Lut, there is a correction of this qualitative imbalance in leukocytes, evidenced by a 43.6% reduction in the total number of leukocytes. Additionally, the qualitative imbalance is corrected with a 37% decrease in neutrophils and a 14% increase in lymphocytes. LUT-Opz exhibited marked immunomodulation in the stomach lining when compared to LUT200; however, Opz had little influence on inflammation, which is in line with its function as a gastric acid secretion suppressor. The evidence from the histologic studies was confirmed by the microscopic evaluation of the gastric mucosa homogenates stained using Wright-Giemsa technique, which similarly demonstrated a significant number of neutrophils in the GU group compared to the control, as well as evident reduction in the number of leukocytes in the Lut-treated groups (Figure 2F).

**Figure 2:**
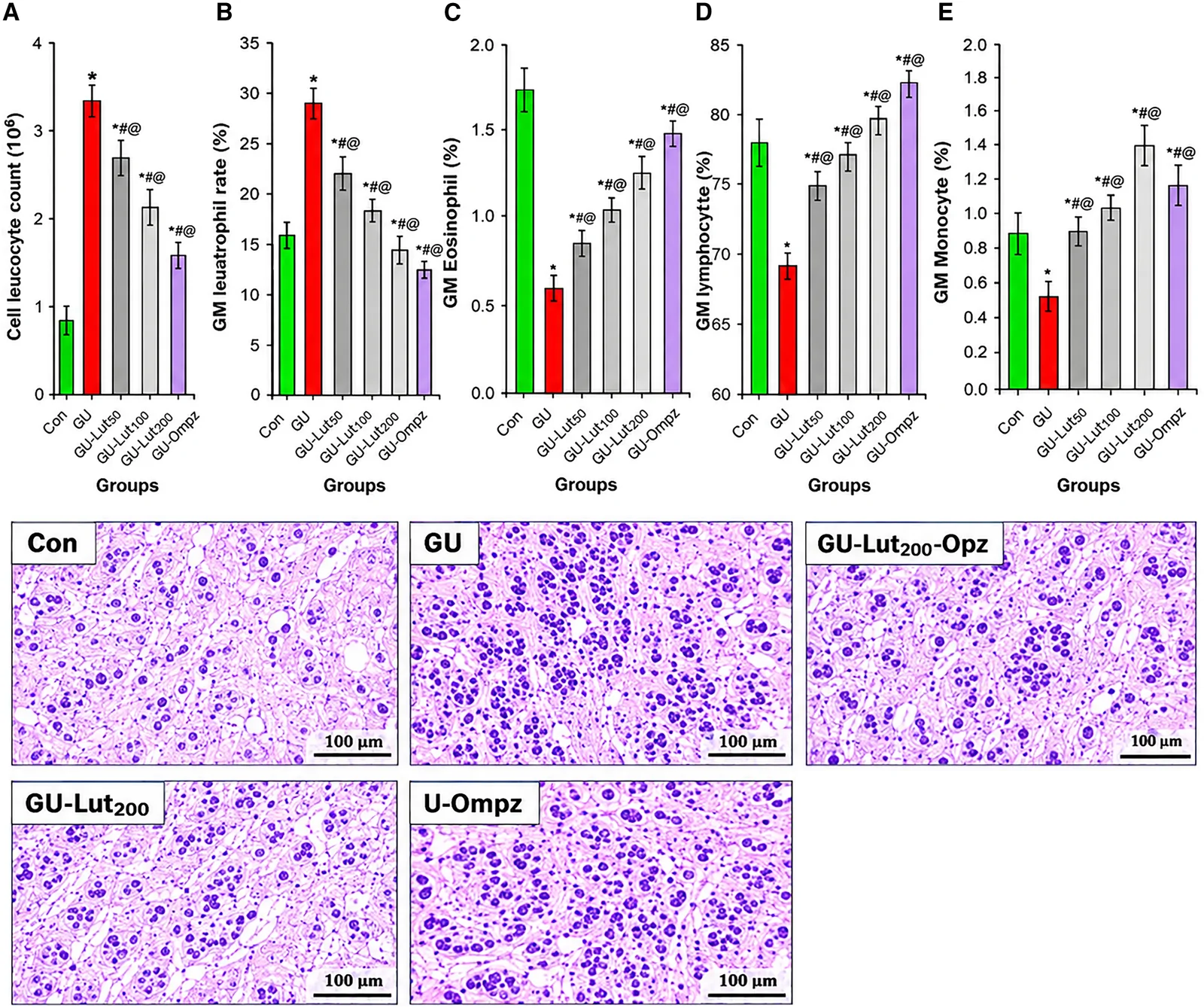
Effect of Lut treatment on total leukocytes (A), NEU (B), EOS (C), LYM (D), and MON (E), with Wright–Giemsa staining (F) showing increased neutrophil infiltration in GU rats and dose-dependent restoration of leukocyte profiles toward normal values after Lut treatment Evaluation of absorbance (A) and FTIR (B) spectra of isolated Lut from chlorella algae: confirmation of effective purification protocol. (A) Total leukocytes. (B) NEU. (C) EOS. (D) LYM. (E) MON. (F) Wright-Giemsa stained picture showing infiltration of leukocytes in the stomach lining. *n* = 6; ∗*p* < 0.05 vs. normal control; ^@^*p* < 0.05 vs. untreated ulcer; ^#^*p* < 0.05 vs. Lut_50_; ^&^*p* < 0.05 vs. Lut_100_; ^α^*p* < 0.05 vs. Lut_200_; ^β^*p* < 0.05 vs. Lut_200_-Opz. Data are represented as mean ± SEM (*n* = 6). Scale bars, 100 μm.

### Effect of Lut on neutrophil infiltration score, NLR, and MPO activity

Ethanol ingestion caused a significant increase in neutrophil counts and infiltration in gastric tissues, evidenced by remarkable elevations in NIS (3A) and NLR (3B), along with a 234% rise in MPO activity (3C) compared to normal rats. In contrast, Lut supplementation reduced and suppressed this infiltration in a dose-dependent manner. For example, administration of Lut at a dose of 200 mg/kg body weight reduced MPO activity by 57% and NIS by 83% compared to untreated ulcerated rats, potentially inhibiting neutrophil infiltration. Furthermore, treatment with 200 mg/kg of Lut normalizes and balances the NLR (Figure 3). Nevertheless, the treatment LUT-Opz showed significant differences in the levels of neutrophil score infiltrate, neutrophil/lymphocyte ratio, and myeloperoxidase activity in comparison with LUT_200_. Opz alone failed to show any effect on the markers of inflammation mentioned above due to its major function as a secretion inhibitor of gastric acid.

**Figure 3:**
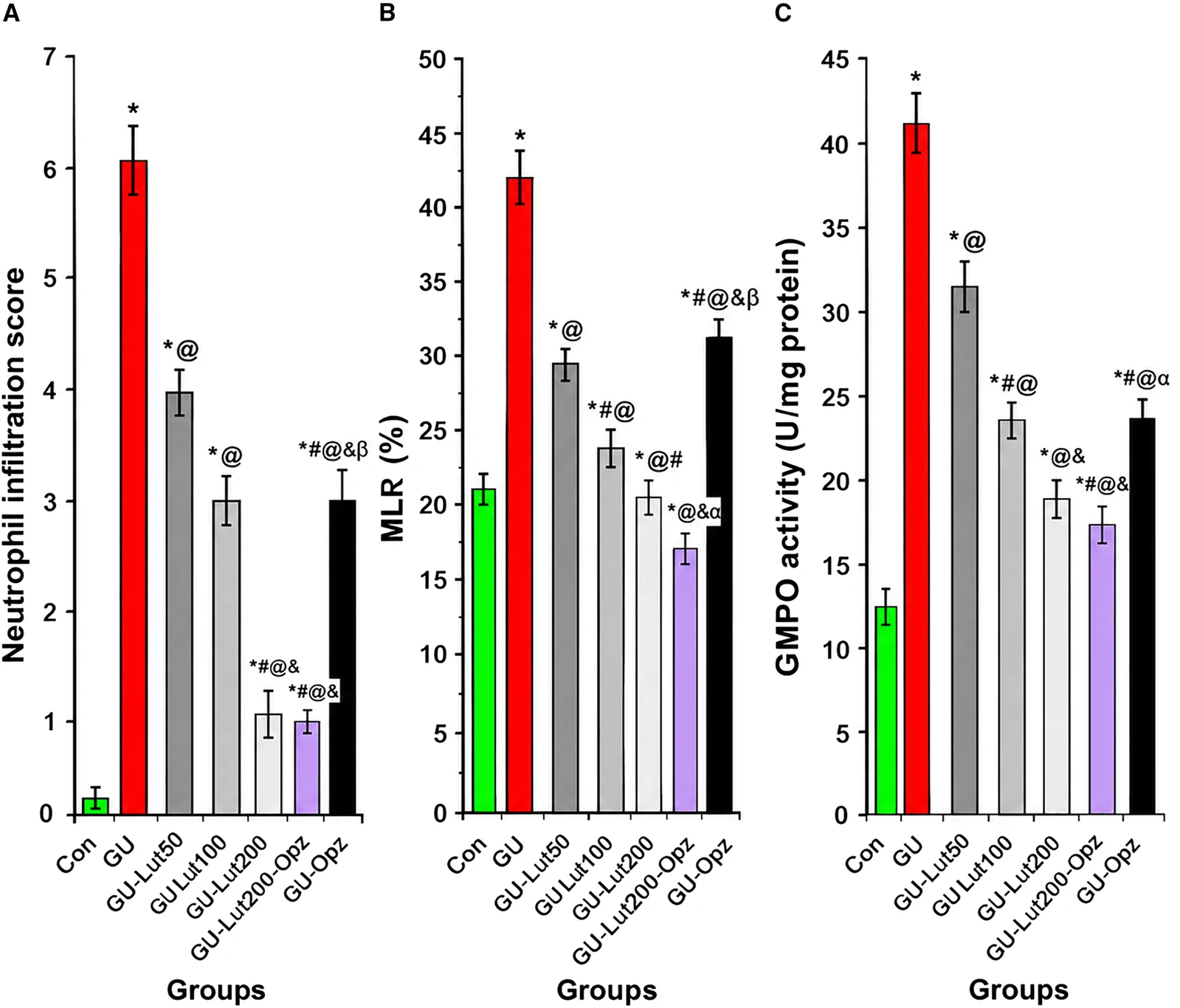
Effect of Lut treatment on neutrophil infiltration in gastric tissue of ulcerated rats (A) NIS. (B) NLR. (C) MPO activity. *n* = 6; ∗*p* < 0.05 vs. normal control; ^@^*p* < 0.05 vs. untreated ulcer; ^#^*p* < 0.05 vs. Lut_50_; ^&^*p* < 0.05 vs. Lut_100_; ^α^p < 0.05 vs. Lut_200_; ^β^p < 0.05 vs. Lut_200_-Opz. Data are represented as mean ± SEM.

### Effect of Lut on COX activity, NO, CRP, and PGE_2_ levels

This study shows that gastric ulcers significantly increase COX activity (A) and PGE2 (B), NO (C), and CRP (D) levels while reducing hexosamine rate in the gastric juice. However, ingestion of Lut by ulcerated rats results in a significant reduction in COX activity and CRP, NO and PGE2, levels in a dose-dependent manner. For instance, treatment with 200 mg/kg of Lut led to a notable decrease in COX activity by 50%; and CRP, NO, and PGE2 levels by 67%, 39%, and 55% compared to untreated ulcerated rats (Figure 4). LUT_200_-Opz combination produced significant effects on the activities of COX, NO, CRP, and PGE_2_ levels, as LUT200, but Opz alone produced limited anti-inflammatory effects.

**Figure 4:**
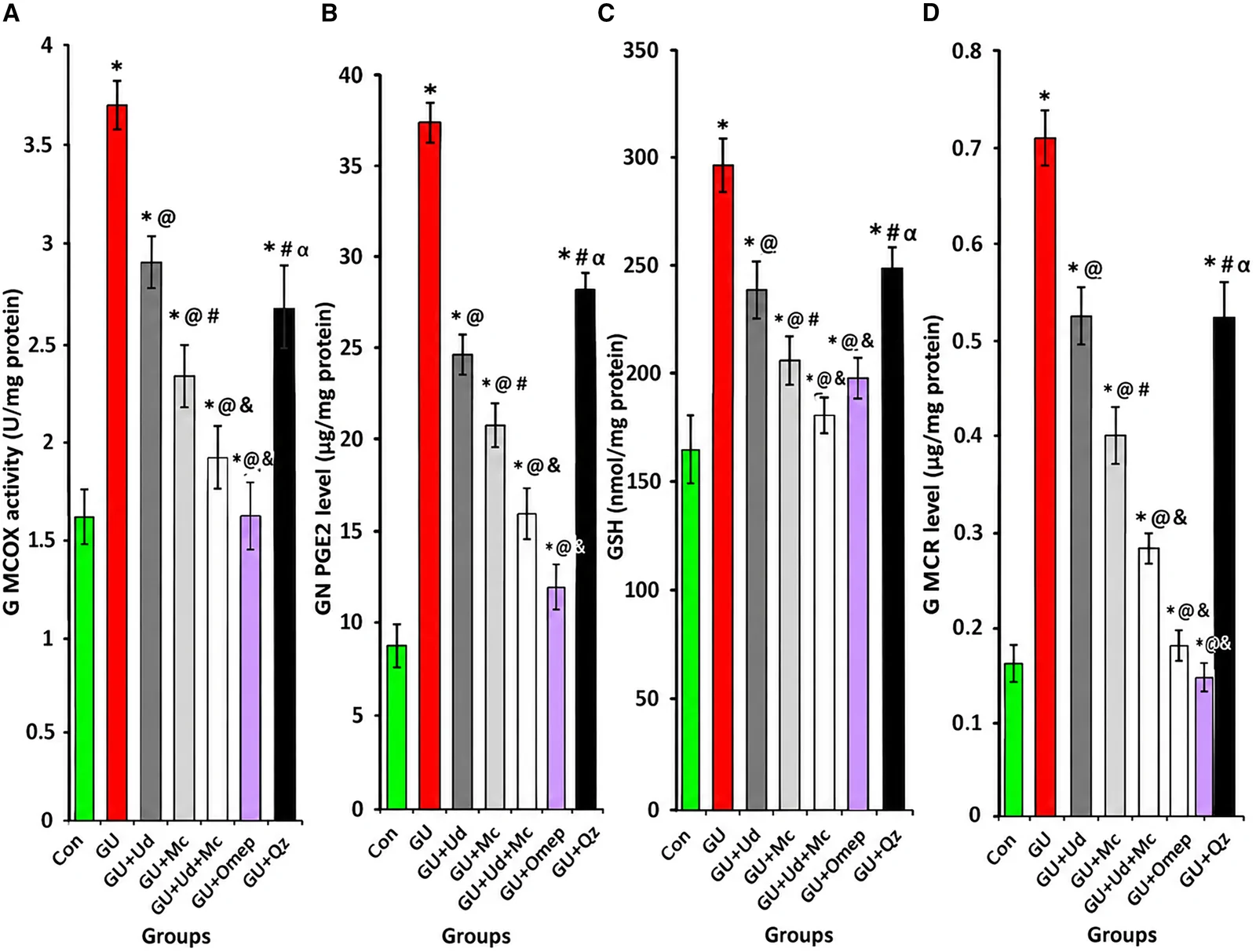
Effect of Lut on inflammatory markers in ulcerated rats (A) COX activity. (B) PGE_2_ levels. (C) NO levels. (D) CRP levels. *n* = 6; ∗*p* < 0.05 vs. normal control; ^@^*p* < 0.05 vs. untreated ulcer; ^#^*p* < 0.05 vs. Lut_50_; ^&^*p* < 0.05 vs. Lut_100_; ^α^p < 0.05 vs. Lut_200_; ^β^p < 0.05 vs. Lut_200_-Opz. Data are represented as mean ± SEM.

### Effect of Lut on oxidative stress in the gastric mucosa: H_2_O_2_ and TBARS levels

Figure 5 shows that oral administration of absoLute ethanol induced severe oxidative stress in gastric juice, as evidenced by a significant increase in H_2_O_2_ (A) and TBARS (B) levels. However, gavage with Lut provided substantial protection against free radicals. As illustrated in Figure 3, both doses of Lut significantly reduced gastric oxidative stress. The reduction in H_2_O_2_ and TBARS levels was more pronounced in the Lut group at a 200 mg dose, with decreases of 69% and 75%, respectively. In the combined Lut-Opz pretreated groups, reductions of 71% and 79%, respectively, were observed compared to untreated ulcerated rats (Figure 5). The combination of LUT_200_-Opz decreased the levels of H_2_O_2_ and TBARS more efficiently compared to LUT_200_, while Opz alone exhibited minimal antioxidant efficacy.

**Figure 5:**
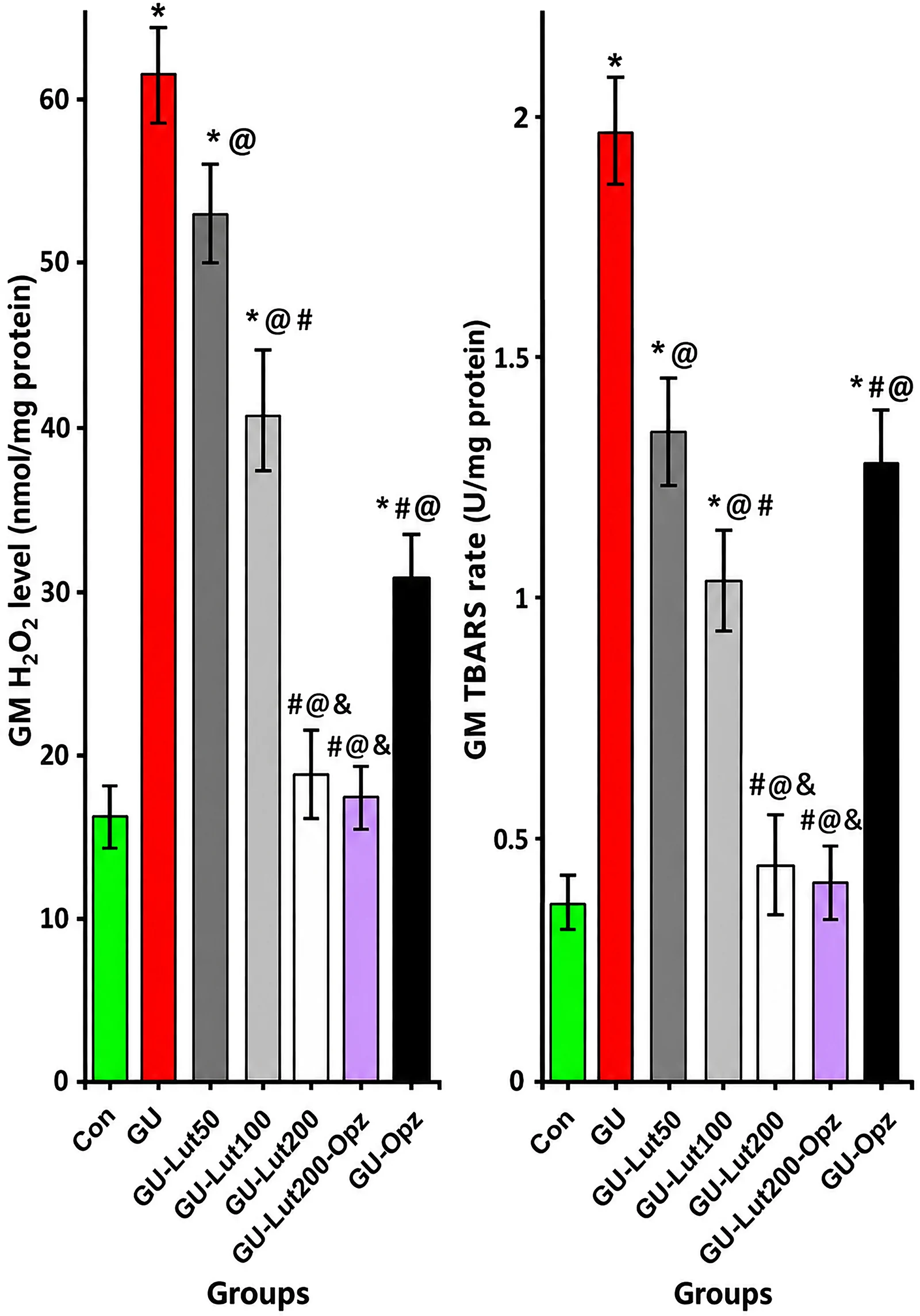
Effect of Lut on oxidative stress markers in gastric mucosa (A) H_2_O_2_ levels. (B) TBARS levels. *n* = 6; ∗*p* < 0.05 vs. normal control; ^@^*p* < 0.05 vs. untreated ulcer; ^#^*p* < 0.05 vs. Lut_50_; ^&^*p* < 0.05 vs. Lut_100_; ^α^p < 0.05 vs. Lut_200_; ^β^p < 0.05 vs. Lut_200_-Opz. Data are represented as mean ± SEM.

### Effect of Lut on gastric secretory function

Our results show that gastric ulcers lead to disruption and dysfunction of gastric function, evidenced by decreases in gastric mucosa (A), hexosamine (B), and mucus (C) contents, as well as increases in pepsin activity (D), pH values, and gastric juice volume (Table 1). In contrast, Lut administration corrected these disturbances and reinforced mucosal protection by significantly increasing protective compounds such as gastric mucosa, hexosamine, and mucus contents, while suppressing pepsin activity, pH levels, and the volume of gastric secretion. (Table 1; Figure 6). The impact of the LUT_200_-Opz association appeared to be more expressed, but not statistically significant, compared with LUT200 on GMBC, hexosamine, and mucus content as well as on pepsin activity. This impact was stronger compared with Opz itself, whose effects were statistically significant and related to its main mechanism of action.

**Table 1:** Effects of gastric ulceration and Lut administration on NO, SOD, CAT, GPx, gastric ulcer area (GUA), ulcer index (UI), curative index (CI), gastric juice volume (GJV), and pepsin activity

	Con	GU	GU-lut_50_	GU-lut_100_	GU-lut_200_	GU-lut_200_-Opz	GU-Opz
GM SOD (U/mg protein)	0.63 ± 0.04	0.21 ± 0.05∗	0.35 ± 0.01∗^@^	0.54 ± 0.03∗^@#^	0.68 ± 0.041^@#&^	0.69 ± 0.02^@#&^	0.37 ± 0.017∗^@&αβ^
GM CAT (U/mg protein)	1.89 ± 0.12	0.59 ± 0.02∗	0.91 ± 0.04∗^@^	1.67 ± 0.09∗^@#^	1.97 ± 0.11^@#&^	1.94 ± 0.12^@#&^	0.98 ± 0.041∗^@&αβ^
GM GPx (U/mg protein)	17.1 ± 2.4	3.12 ± 0.12∗	8.97 ± 0.43∗^@^	14.3 ± 0.9∗^@#^	18.1 ± 1.1^@#&^	18.6 ± 1.3^@#&^	10.1 ± 0.61∗^@&αβ^
pH	6.71 ± 0.51	4.03 ± 0.25∗	4.81 ± 0.26∗^@^	6.13 ± 0.37∗^@#^	6.64 ± 0.32^@#&^	6.83 ± 0.29^@#&^	6.12 ± 0.23^&αβ^
GUA (mm^2^)	00	791 ± 28∗	561 ± 26∗^@^	369 ± 21∗^@#^	151.2 ± 7.9∗^@#&^	69 ± 24∗^@#&α^	73.2 ± 6.3∗^@#α^
UI (%)	00	77.8 ± 3.1∗	55.3 ± 2.3∗^@^	36.4 ± 2.1∗^@#^	14.9 ± 0.9∗^@#&^	8.29 ± 0.31∗^@#&α^	9.25 ± 0.41∗^@#&α^
CI (%)	100	22.2 ± 1.1∗	54.7 ± 3.1∗^@^	63.6 ± 3.4∗^@#^	75.1 ± 3.1∗^@#&^	91.59 ± 3.7∗^@#&α^	90.7 ± 3.9∗^@#&α^
GJV (mL)	1.59 ± 0.08	2.69 ± 1.3∗	2.27 ± 0.13∗^@^	2.1 ± 0.13∗^@^	1.89 ± 0.15∗^@#&^	1.47 ± 0.19∗^@#&α^	1.60 ± 0.31^@#&α^
GS Pepsin activity (U/L)	3,016 ± 167	1,057 ± 49∗	1,578 ± 68∗^@^	2,179 ± 113∗^@#^	2,697 ± 107^@#&^	2,987 ± 121∗^@#&α^	2,897 ± 103^@#&α^

Gastric ulcer significantly increased NO levels and decreased SOD, CAT, and GPx activities, whereas LUT administration dose-dependently reversed these alterations. ∗*p* < 0.05 compared to the healthy control group. ^@^*p* < 0.05 compared to the untreated ulcer group. ^#^*p* < 0.05 compared to the Lut_50_-ulcer treated group. ^&^*p* < 0.05 compared to the Lut_100_-ulcer treated group. ^α^p < 0.05 compared to the Lut_200_-ulcer treated group. ^β^p < 0.05 compared to the with Lut_200_—Opz-ulcer treated group.

**Figure 6:**
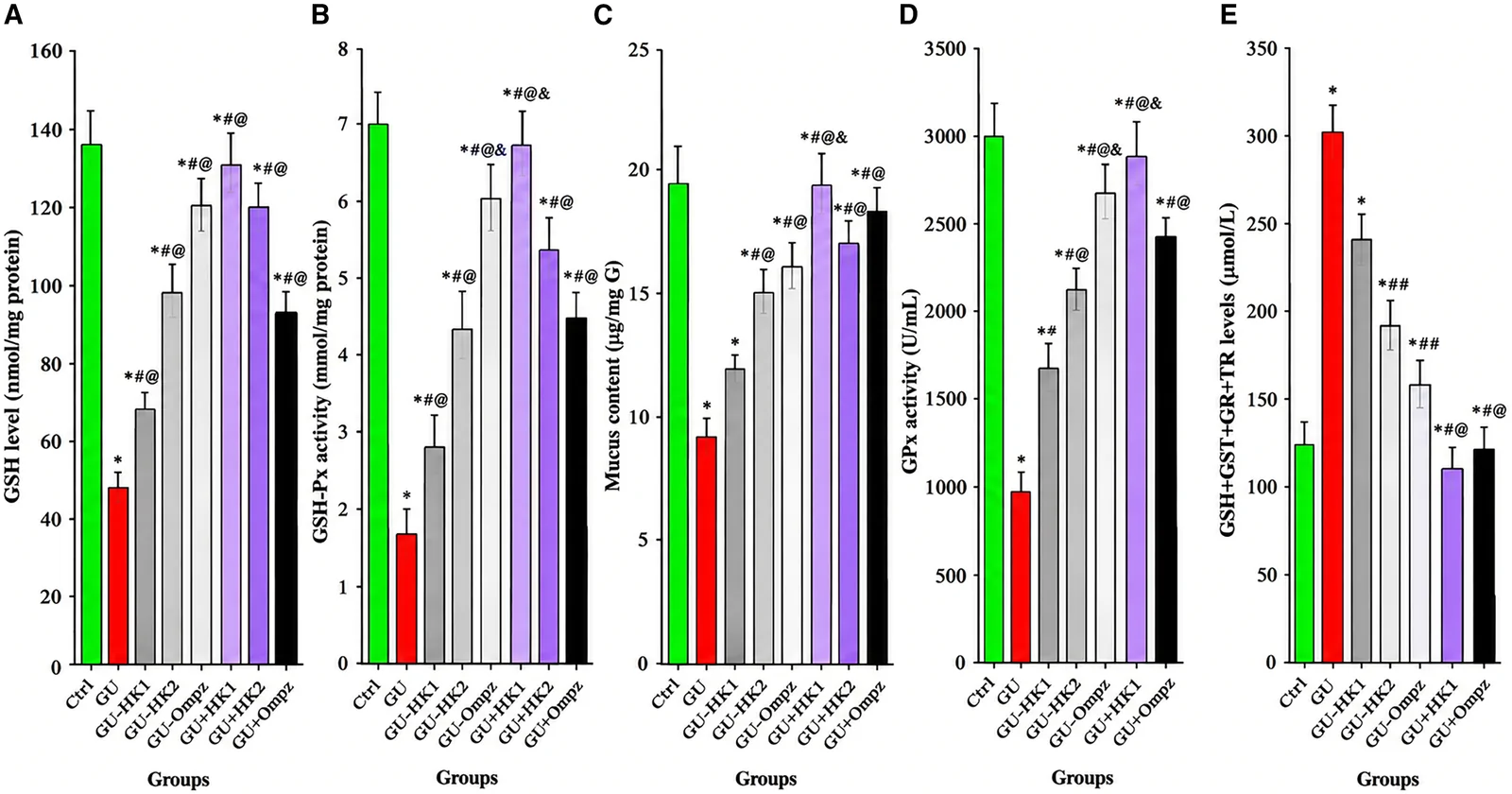
Effect of Lut on gastric protective factors in ulcerated rats (A) GMBC. (B) Hexosamine. (C) Mucus. (D) Pepsin activity. (E) H^+^/K^+^-ATPase. *n* = 6; ∗*p* < 0.05 vs. normal control; ^@^*p* < 0.05 vs. untreated ulcer; ^#^*p* < 0.05 vs. Lut_50_; ^&^*p* < 0.05 vs. Lut_100_; ^α^p < 0.05 vs. Lut_200_; ^β^p < 0.05 vs. Lut_200_-Opz. Data are represented as mean ± SEM.

### Effect of LUT on H^+^/K^+^-ATPase activity

Ulcers caused by ethanol administration led to significant increase in H^+^/K^+^-ATPase activity in the gastric tissue by 140% when compared to the control group. Administration of LUT dose dependently inhibit the H^+^/K^+^-ATPase activity in ulcerated rats in the amount of 15.3%, 28.6%, and 43.2% when LUT was administered at the doses of 50, 100, and 200 mg/kg, respectively, when compared to the ulcerated group. The LUT200-Opz combination produced a marked reduction of 59.5%, restoring H^+^/K^+^-ATPase activity close to control values. Opz alone decreased H^+^/K^+^-ATPase activity by 55.1%, confirming its inhibitory effect on gastric proton pump activity (Figure 6E).

### Effect of ethanol and Lut on macroscopic gastric mucosa architecture

Control rats exhibited a normal gastric mucosa structure with typical mucosal thickening. In contrast, the ethanol-treated group showed severe tissue damage, including dark red submucosal hemorrhagic streaks of varying sizes and increased mucosal thickening (GU). This was confirmed by a significant increase in GUA and UI compared to normal rats (Table 1). However, rats pretreated with Lut before ethanol exposure showed excellent protection, displaying mostly normal pink mucosa with minimal thickening and few noticeable hemorrhages or congestion, depending on the dose administered (GU-Lut_50_, GU-Lut_100,_ GU-Lut_150_, and GU-Lut_200_) In fact, pretreatment of ulcerated rats with Lut potentially reduced GUA and UI by about 80%, with results nearly comparable to those seen in the Opz group (Figure 7). The Lut_200_-Opz combination exhibited a more effective protective effect against GUA compared with Lut_200_ and Opz alone.

**Figure 7:**
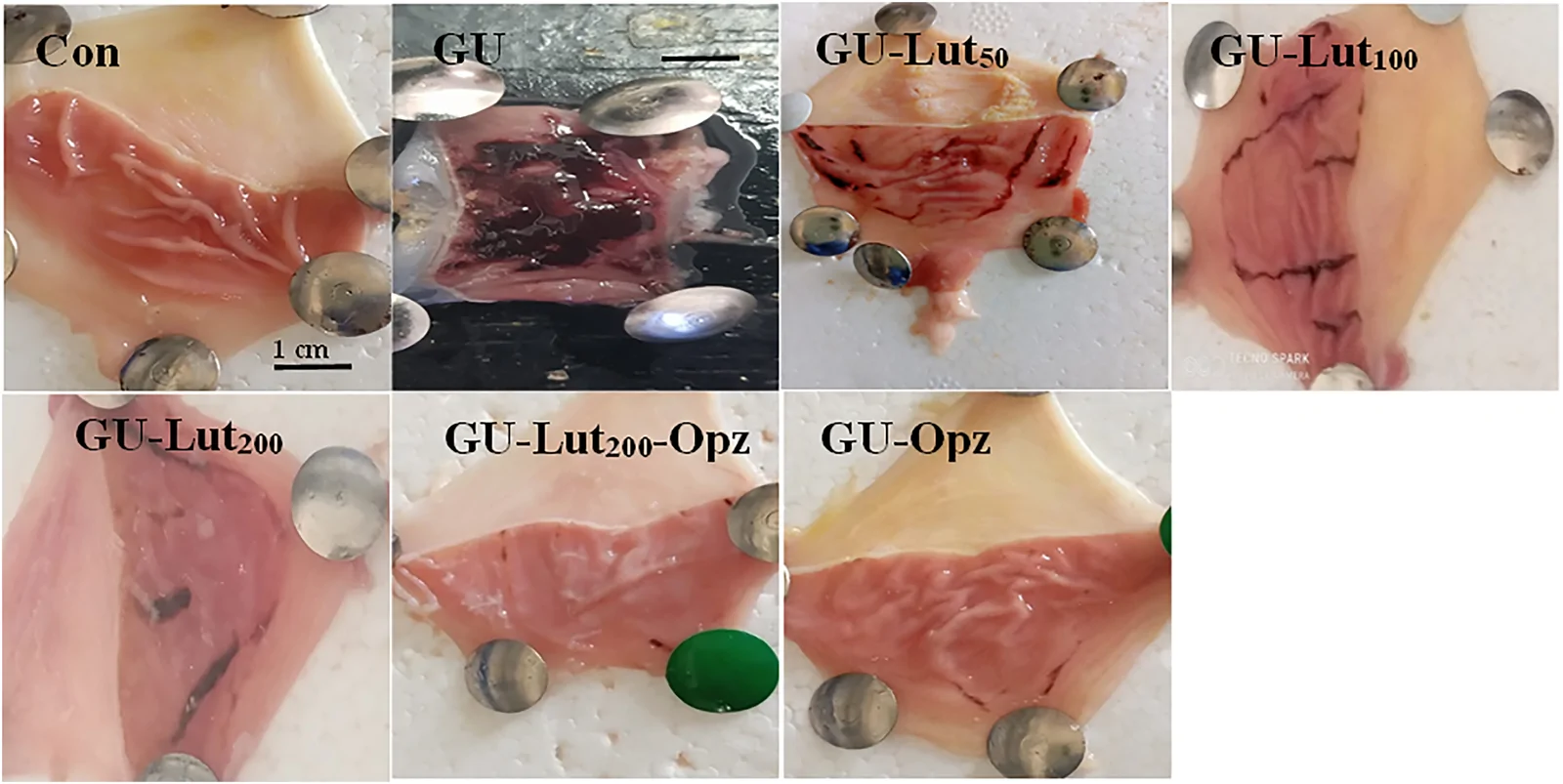
Effect of Lut on macroscopic gastric lesions The scale bars represent 1 cm.

### Effect of Lut on gastric histopathological alterations

Hematoxylin and eosin staining of the gastric mucosa histology showed that there were no lesions and inflammation infiltrating in the control group (Con). However, the stomach ulcer group (GU) presented with severe epithelial erosion, tissue necrosis, submucosal edema, and inflammation. Treatment of the GU group with 200 mg/kg luteolin and omeprazole (GU-Lut_200_-Opz) almost resulted in complete recovery to normal gastric architecture with well-formed glands and little inflammation. The GU group receiving treatment with 200 mg/kg luteolin alone (GU-Lut_200_) was also significantly improved, even though there was some glandular disorganization and inflammation. The GU group receiving treatment with omeprazole (GU-Ompz) partially recovered its mucosa (Figure 8).

**Figure 8:**
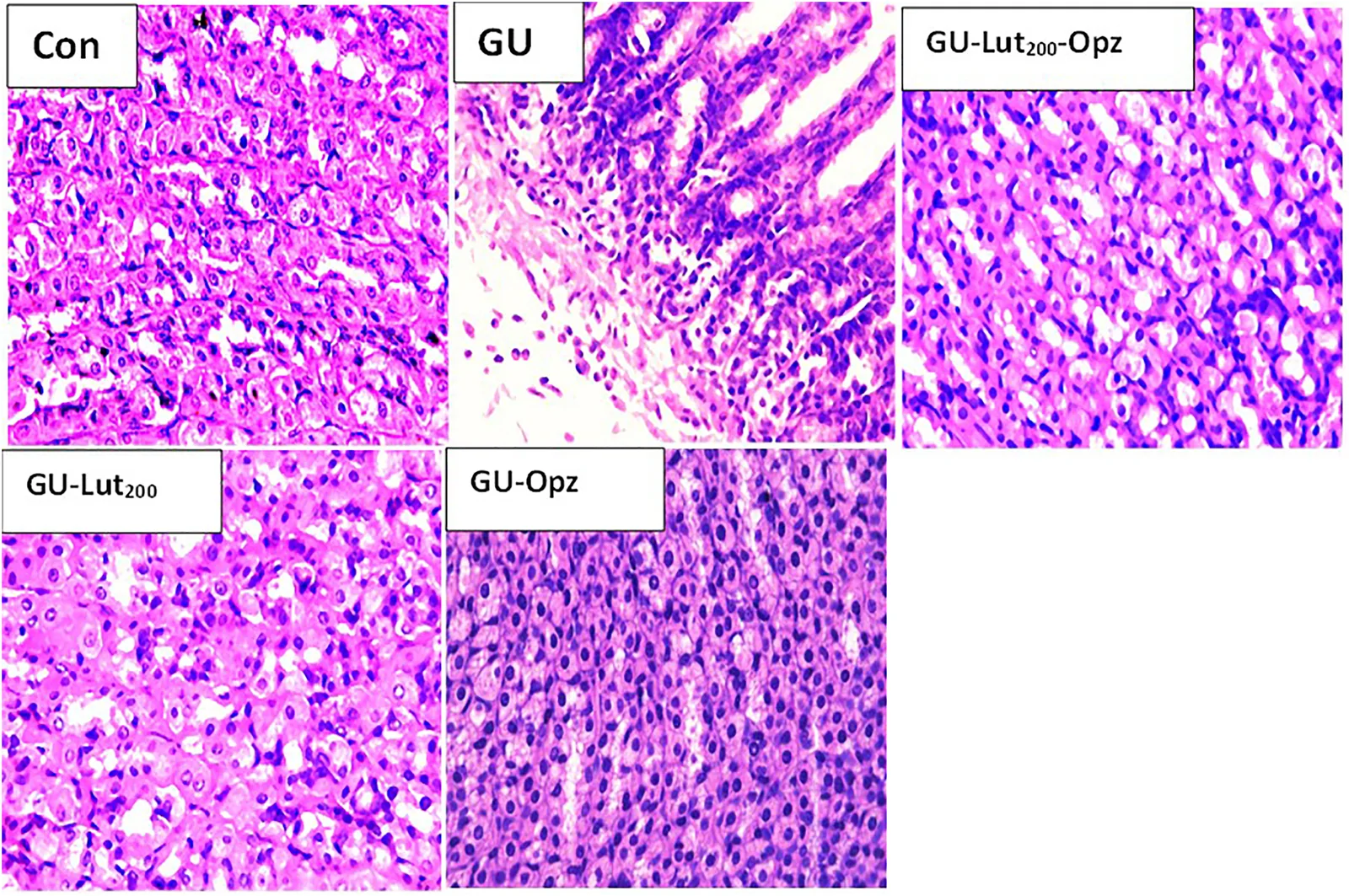
Effect of Lut on histopathological alterations of the gastric mucosa in ulcerated rats H&E staining. Scale bars 50 μm.

## Discussion

According to scientific studies, excessive alcohol consumption is associated with numerous adverse effects, including the development of gastric ulcers. After ingestion, ethanol rapidly penetrates the gastric mucosa, causing the recruitment and infiltration of leukocytes into the stomach tissues.^25^^,^^26^ This process subsequently triggers neutrophil-driven inflammatory reactions, characterized by the overproduction of reactive oxygen species, activation of key inflammatory enzymes, and excessive release of pro-inflammatory cytokines. These responses lead to significant gastric tissue damage. The resulting lesions closely resemble human alcohol-related gastric injuries, presenting as submucosal edema, bleeding, epithelial cell loss, and infiltration of inflammatory cells.^27^

Regarding the characterization of isolated LUT, spectrophotometric and FTIR measurements indicated the efficiency of the LUT extraction process and its structural preservation. High purification rate (88%) compared to a commercial LUT sample confirms the effectiveness of the extraction method and is in good agreement with literature data on purity of lutein obtained from Chlorella algae (85%–98%).^28^^,^^29^ Absorption maxima at 445 and 475 nm prove the presence of characteristic profile of carotenoids in LUT, whereas FTIR spectroscopy showed the presence of typical O–H, C–H, and C=C bonds.^30^^,^^31^

In this context, Administration of ethanol caused immunological dysregulation of the stomach, where there was an increased neutrophilic activity, as indicated by the increased number of leukocytes, NLR, NIS, and MPO levels (Figures 2 and 3). This is in agreement with Li et al.^32^ who found out that there is an increase in gastric MPO activity after ethanol administration, indicating neutrophilic accumulation and inflammation. This inflammatory process led to increased production of NO, oxidative stress (H_2_O_2_ and TBARS), and reduced antioxidant activities (SOD, CAT, and GPx) (Figures 4 and 5). There was thus impairment of the protective mechanisms of the gastric mucosa through reduction of the mucus and hexosamine levels, leading to increased ulcer area and ulcer index (Table 1; Figure 6). These observations are consistent with previous reports showing that ethanol disrupts gastric microcirculation and blood flow.^33^ Moreover, our findings align with those of Mahmoud and Abd El-Ghffar^34^; Selim et al.^35^ and Syed et al.^36^ who demonstrated that gastric ulceration is closely associated with inflammation and oxidative stress.

In view of the essential role of neutrophils migration, inflammation, and oxidative stress in developing the lesions in the gastric mucosa, the drawbacks of the conventional treatment approach underscore the need for safe approaches. Antioxidant, anti-inflammatory, and gastroprotective compounds including Lut may be considered potential candidates due to their antioxidant properties leading to reducing oxidative stress and production of reactive oxygen species. This study demonstrates the gastroprotective property of lutein against ethanol-induced gastric ulcers by inhibiting neutrophils migration due to reduced NIS, NLR, and MPO activity (Figures 2 and 3), as well as reducing gastric inflammation via lowering COX, NO, CRP, and PGE_2_ levels (Figure 4). The effect was also validated through histopathological analysis, which observed a significantly decreased number of neutrophils, tissue necrosis, and damage to the mucosa of the stomach of rats treated with Lut at 200 mg/kg body weight. Histological studies using hematoxylin and eosin staining observed that the structure of the gastric mucosa of the control group (Con) was intact with no presence of lesions or inflammatory cells, while ulcerated rats (GU) displayed extensive tissue necrosis and erosion. The above results agree with Meurer et al.,^37^ which reported that the use of Lut on rich plants resulted in reduced MPO level in ulcerated rats thus causing reduced inflammation via inhibition of inflammatory cell migration. Similarly, Hadad and Levy^38^ demonstrated that Lut significantly inhibited neutrophil recruitment in rats with LPS-induced inflammation. Similarly, Ahn and Kim,^39^ noted that Lut inhibits neutrophilic migration in LPS induced inflammation, whereas Chen et al.^40^ have shown that Lut results in attenuation of inflammation via regulation of TLR4/NLRP3/caspase-1 pathway, while Xu et al.^41^ ave discovered that Lut causes preservation of intestinal barrier function during DSS induced colitis through inhibition of NF-κB and reduced expression of TNF-α, iNOS, NLRP3, and IL-1β.

Oxidative stress develops due to the imbalance between the generation of ROS and antioxidants. The generation of ROS in the formation of ulcers is connected to the invasion of inflammatory cells in the stomach mucosa. Consistent with this process, our research showed that Lut decreased the invasion of leukocytes, which was demonstrated by the decrease of MPO activity causing the reduction in H_2_O_2_ and TBARS production and consequently less oxidative stress (Figure 5). Also, Lut increased antioxidant defense mechanisms, including the improvement in SOD, CAT, and GPX activities (Table 1). These results are consistent with Meurer et al.,^42^ who showed that Lut prevents DSS-induced colitis through its antioxidant action and antioxidant enzyme activity restoration. n a similar vein, Lut enhanced gastric defense mechanisms, such as mucus, gastric mucosa barrier, and hexosamine concentrations (Figure 6), thus ensuring mucosal integrity. There is scant literature on Lut in relation to gastric ulcers. Meurer et al.^43^ have demonstrated the anti-inflammatory property of the Lut-rich extracts due to their low MPO activities and inhibition of pro-inflammatory mediators, consistent with the anti-inflammatory effect of carotenoids. For example, the study by Bahrini et al.^44^ showed that astaxanthin attenuates gastric ulceration by reducing inflammation and promoting mucus production. These findings support the potential role of carotenoids, including Lut, in gastric mucosal protection.

As seen in this study, Lut-induced mechanisms such as the prevention of inflammation, increase in the levels of antioxidants, and boosting of gastric defense system through enhanced mucus secretion all work together in protecting the gastric cells by lowering the levels of GUA and UI (Table 1). Despite ethanol causing acute gastric ulcers due to vasculature injury and necrosis of epithelial cells, administration of Lut (50, 100, and 200 mg/kg) led to a considerable reduction in ulcers. The chosen concentrations of Lut were based on a prior toxicity evaluation of the substance, making them safe for future experiments. Moreover, this protection was evidenced by functional studies of the stomach, in which an improvement in mucus, pH, gastric juice volume, and decrease in pepsin activity (Table 1; Figure 7) were observed.^45^^,^^46^ The pepsin and H^+^/K^+^-ATPase are the enzymes responsible for gastric acidity and development of ulcers, so their inhibition through Lut can play an important role in maintaining gastric mucosal integrity. Besides, Lut promoted a dose-dependent increase in the mucus and histamine secretion with the maximum effect at the combination of Lut with omeprazole. Plant compounds have also been shown to raise the amounts of hexosamine and hexose, thus improving the mucosal barrier and mucus secretion.^47^ In total, gastroprotection mediated by Lut is due to various mechanisms, such as inhibition of inflammation, antioxidation, stimulation of protective factors in the mucosa, and supression of gastric acid secretion via pepsin and H^+^/K^+^-ATPase activation.

Regarding the combined therapy, the results have shown that LUT_200_ combined with Opz demonstrated synergistic gastroprotection against the effects of ethanol-induced damage to the gastric tissue as opposed to the administration of individual treatments. LUT_200_ was found to exert an impact on the ulcerative process mainly via targeting the processes of inflammation and oxidation, since it was able to inhibit inflammatory mediators, prevent neutrophils’ migration, reduce the production of reactive oxygen species and restore the functions of antioxidant defense system. On the other hand, Opz was found to affect the gastric acid secretory mechanism via the suppression of the activity of H^+^/K^+^-ATPase and subsequent decrease of gastric acidity. Thus, the co-administration of LUT_200_ and Opz allowed targeting multiple mechanisms implicated in the pathogenesis of gastric ulcers. In turn, Opz alone had no significant effect on inflammatory and oxidative stress parameters due to its acid-suppressive action. Further investigations through istology demonstrated the distinct difference between GU-Lut_200_-Opz and GU-Opz treatments. GU-Lut_200_-Opz led to almost complete restoration of the stomach lining with organized glands, nearly normal architecture of the lining, and very little inflammatory cells in the lining. This is comparable to the control group. On the other hand, GU-Opz led to partial recovery of the stomach lining, where there was some presence of inflammatory cells and not complete organization of the glands.

In conclusion, this study highlights the gastroprotective effects of Lut against ethanol-induced gastric ulcers in rats. These beneficial effects appear to result from multiple mechanisms. Lut significantly reduced the infiltration of neutrophils and lymphocytes into the gastric juice, as evidenced by cell counts and decreased MPO activity. It also suppressed gastric inflammation, enhanced the antioxidant defense system by inducing antioxidant enzymes (SOD, CAT, GPx), and consequently reduced levels of H_2_O_2_ and TBARS. Furthermore, it inhibited key enzymes involved in ulcer formation and gastric acidity, such as pepsin. These protective effects were confirmed by macroscopic analysis of gastric tissues, which showed a dose-dependent improvement, with the highest protection observed at 200 mg/kg body weight. Additionally, this study demonstrated that the co-administration of Lut and omeprazole resulted in synergistic effects, offering the most effective protection against gastric ulcers. These protective effects were further supported by macroscopic examination of gastric tissues.

### Limitations of the study

There are some limitations associated with this study. For one thing, experiments were carried out in an acute ethanol-induced gastric ulcer in rats. However, there is a high probability that such a model cannot represent the complexity of a chronic or human gastric disease. Thus, there is a need for further studies to confirm the findings using other types of experimental models. Another limitation is that the gastric stability, absorption, metabolism, distribution, and bioavailability of lutein have not been studied in detail. Pharmacokinetic research is necessary to determine the relation between doses and biological activities. Moreover, despite the fact that lutein regulates inflammatory mediators, oxidative stress, antioxidant enzymes, leukocyte count, and H^+^/K^+^ATPase activity, no investigation was done regarding the molecular mechanisms of this effect. Relevant studies should be done to investigate signaling mechanisms. Finally, further dose-response studies, safety studies, and lutein omeprazole interaction studies are needed. Moreover, only male rats have been considered for this experiment. Hence, it is impossible to know whether sex may affect the gastroprotective properties of lutein. Additional experiments with both males and females would shed more light on this issue.

## Resource availability

### Lead contact

Further information and requests for resources and reagents should be directed to and will be fulfilled by the lead contact, Insaf Bahrini (bahriniinsaf4@gmail.com).

### Materials availability

This study did not generate new unique reagents. All materials generated in this study are listed in the key resources table.

### Data and code availability

• All data in this study can be accessed from the respective author upon request.
• There is no original code reported in the study.
• Any other information pertaining to the data in this study can be obtained from the author upon request.

## Acknowledgments

This research has been funded by Sientific Research Deanship at University of Ha’il - Saudi Arabia through project number RG-24 073.

## Author contributions

Conceptualization, I.B. and M.A.; methodology, I.B. and M.A.; investigation, I.B. and M.A.; visualization, I.B.; supervision, M.A.; writing – original draft, I.B.; writing – review and editing, I.B. and M.A. All authors have reviewed and approved the final version of the manuscript.

## Declaration of interests

The authors declare no competing interests.

## Declaration of generative AI and AI-assisted technologies in the writing process

No generative AI and AI-assisted tools were employed in data analysis, content generation, or any other aspects of the study, except for grammar and spelling corrections.

## STAR★Methods

### Key resources table

**Table:** 

REAGENT or RESOURCE	SOURCE	IDENTIFIER
**Chemicals, peptides, and recombinant proteins**		

Lutein standard	Sigma-Aldrich	Cat# PHR1699
Potassium hydroxide (KOH)	Sigma-Aldrich	Cat# 221473
Ascorbic acid	Sigma-Aldrich	Cat# A92902
Dichloromethane	Sigma-Aldrich	Cat# 270997
Absolute ethanol	Sigma-Aldrich	Cat# 24102
Methanol, HPLC grade	Sigma-Aldrich	Cat# 34860
Acetonitrile, HPLC grade	Sigma-Aldrich	Cat# 34851
Trichloroacetic acid (TCA)	Sigma-Aldrich	Cat# T6399
Thiobarbituric acid (TBA)	Sigma-Aldrich	Cat# T5500
Glacial acetic acid	Sigma-Aldrich	Cat# 320099
Hydrochloric acid (HCl)	Sigma-Aldrich	Cat# 320331
Sulfuric acid (H_2_SO_4_)	Sigma-Aldrich	Cat# 258105
Potassium iodide (KI)	Sigma-Aldrich	Cat# 221945
Ammonium molybdate	Sigma-Aldrich	Cat# 277908
Pyrogallol	Sigma-Aldrich	Cat# 254002
Coomassie Brilliant Blue	Sigma-Aldrich	Cat# 27815
N,N,N′,N′-tetramethyl-*p*-phenylenediamine	Sigma-Aldrich	Cat# T7394
*o*-Dianisidine	Sigma-Aldrich	Cat# D3252
Sulfanilamide	Sigma-Aldrich	Cat# S9251
N-(1-Naphthyl)ethylenediamine dihydrochloride (NED)	Sigma-Aldrich	Cat# N9125
Alcian blue	Sigma-Aldrich	Cat# A5268
Ehrlich’s reagent	Sigma-Aldrich	Cat# 156477
Hematoxylin	Sigma-Aldrich	Cat# H9627
Eosin	Sigma-Aldrich	Cat# E4009
Formaldehyde	Sigma-Aldrich	Cat# F8775
Hydrogen peroxide (H_2_O_2_)	Sigma-Aldrich	Cat# H1009
Glutathione	Sigma-Aldrich	Cat# G4251
Cumene hydroperoxide	Sigma-Aldrich	Cat# 247502
Indomethacin	Sigma-Aldrich	Cat# I7378

**Critical commercial assays**		

CRP assay kit	Biolabo, France	Cat# CRP620
Protein assay kit	Biolabo, France	Ref. 97016
PGE_2_ ELISA kit	Elabscience	Cat# E-EL-0034c

**Experimental models: organisms/strains**		

Male Wistar rats	State-certified breeder	N/A
Algae source	N/A	N/A
*Chlorella vulgaris*	Bioalgae Company, Tunisia	N/A

**Software and algorithms**		

StatView Version 5.0	SAS Institute Inc., Cary, NC, USA	N/A
ImageJ	National Institutes of Health	N/A

**Other**		

Percoll	GE Healthcare	Catalog #17-0891-01
Collagenase type E	Sigma-Aldrich	Catalog #C2674
Wright–Giemsa stain	Sigma-Aldrich	Catalog #WG80
C18 reversed-phase column	Agilent	Catalog #A2006050X046
FTIR 2000 spectrophotometer	PerkinElmer	N/A
UV-visible spectrophotometer	PerkinElmer	N/A
Neubauer hemocytometer	Marienfeld	Catalog #650030

### Experimental model and study participant details

#### Animals

Adult male *Wistar* rats (*Rattus norvegicus*) aged 9 weeks with body weight of 137–145 g were obtained from a breeding firm authorized by the state. The rats were kept in groups of six rats per cage in a conventional polystyrene cage of dimensions 30 cm × 30 cm x 10 cm (length x width x height) in an environment maintained at 23.5°C of temperature, 55 ± 5% of relative humidity, and a light-dark cycle of 12 h. The experiments were conducted according to the accepted protocols and ethics concerning laboratory animals. The protocol of the experiments was approved by the Institutional Animal Ethics Committee (Animal Ethics Approval number TNAPO-06-B-071; TNC#2024-3122).∗∗ Only adult male rats were used for this experiment.

#### Rat oral ulcer model

Gastric ulcers were induced by administering 5 mL/kg of absolute ethanol via gastric gavage. A total number of 42 adult male Wistar rats were used and grouped into seven groups (*n* = 6 per group. Group 1 was the normal control group without gastric ulcers (Con). Group 2 consisted of ulcerated control rats (GU). Groups 3, 4, and 5 consisted of ulcerated rats treated with Lut at doses of 50, 100, and _200_ mg/kg body weight, respectively, and were designated GU-Lut_50_, GU-Lut_100_, and GU-Lut_200_^48^^,^^49^ respectively. Group 6 consisted of ulcerated rats treated with a combination of Lut (200 mg/kg body weight) and Opz./kg^9^^,^^50^ (20 mg/kg body weight) and was designated GU-Lut_200_-and named **GU-Lut**_**200**_**-Opz**. Group 7 consisted of ulcerated rats treated with omeprazole alone (20 mg/kg body weight) and was designated GU-Opz. Three hours after ethanol administration, or 4 h after Lut treatment by gastric gavage route,^9^^,^^50^ the rats were anesthetized with an intraperitoneal injection of sodium pentobarbital at a dose of 50 mg/kg bw, then sacrificed. The stomachs of all rats were opened along the greater curvature, rinsed with normal saline, and pinned flat for ulcer scoring.

### Method details

#### Chlorella algae lut extraction

##### Isolation of lut

Lut was purified from the *Chlorella vulgaris* (purchased from Bioalgae Company, Tunisia) following the procedure described by Aman et al.^51^ In brief, 100 g of dried Chlorella algae powder was dissolved in 250 mL of a 10.0 M KOH solution containing 2.5% ascorbic acid. The mixture was incubated at 60°C for 10 min and then allowed to cool. To isolate Lut, 50 mL of dichloromethane was added to the mixture, followed by centrifugation at 10,000 g for 15 min to recover the supernatant. The obtained extract was washed with 30% ethanol until the aqueous phase became nearly colorless and reached a neutral pH. The phases were separated, and the organic layer was dried by rotary evaporation at 40°C. The dried residue was dissolved in 85% ethanol. Following the dissolution of Lut in 85% ethanol, the Lut n precipitated as the ethanol evaporated. After Lut precipitated, it was filtered and lyophilized until it was completely dry.

##### HPLC analysis of lut

Using HPLC in accordance with Li et al.^52^ technique, the purity of the extracted Lut was ascertained. A reversed-phase C18 column (4.6 × 250 mm, 5 μm particle size) was used for the analysis, and a mobile phase consisting of methanol, dichloromethane, acetonitrile, and water (80:10:5:5, v/v/v/v) was used. The flow rate was set at 1.0 mL/min, the column temperature was maintained at 25 °C, and the detection wavelength was 450 nm. By comparing the peak areas to a standard, the Lut content was measured, and the yield was computed using the Lut content of the chlorella powder.

##### Evaluation de lut purity

For the determination of the purity of the isolated Lut, two solutions were prepared by dissolving 50 μg of either the isolated Lut or the standard (Sigma-Aldrich, ref. 07168) in 5 mL of ethanol, followed by thorough homogenization. The absorbance of each solution was measured at 445 nm. The purity percentage was calculated using the following formula: % purity = (absorbance isolated Lut n/absorbance standard Lut)∗100.

##### Lut spectral analysis

One milligram of pure Lut was dissolved in one milliliter of dichloromethane for spectral analysis. The sample was scanned to determine the absorbance maxima within the 400–700 nm wavelength range, with dichloromethane used as a reference. The spectral characterization was performed using a UV-visible spectrophotometer.

##### Fourier transform infrared spectroscopy (FTIR) analysis

FTIR was used to investigate the pure Lut to identify the chemical based on its functional groups. Using an FTIR 2000 spectrophotometer, the FTIR analysis was carried out over a spectrum range of 4000–450 cm^−1^.

#### Quantitative and qualitative assessment of leukocytes in gastric mucosa

Following the induction of gastric ulcers, the stomachs were collected, and the gastric mucosa was carefully isolated. To isolate immune cells from the gastric mucosa while preserving their structural integrity, the epithelial layer was first removed by repeated washes with an EDTA/DTT buffer (1–5 mM) at 37 °C under gentle agitation, allowing the release of epithelial cells while minimizing damage to leukocytes residing in the lamina propria.^53^ The remaining tissue was then subjected to enzymatic digestion using collagenase type E (0.5–1 mg/mL) at 37 °C for 30–60 min with gentle agitation, thereby reducing the need for excessive mechanical dissociation and limiting cell damage.^54^ To preserve cell viability, prolonged enzymatic digestion was avoided, as excessive incubation may compromise cell integrity.^55^ Following collection of gastric mucosa, the tissue was gently homogenized to obtain a cellular suspension. The resulting cell suspension was filtered through a 100 μm cell strainer to remove tissue debris and undissociated aggregates, followed by centrifugation at 350 × g for 5 min at 4 °C. The resulting cell suspension was then carefully layered onto a discontinuous Percoll density gradient consisting of 40% and 70% (v/v) Percoll (GE Healthcare) prepared in PBS. The samples were centrifuged at 600 × g for 20 min at room temperature without applying the brake, allowing enrichment of the leukocyte fraction while minimizing contamination by epithelial cells and tissue debris.^53^^,^^56^ The cell fraction at the 40% and/70% Percoll interface was carefully collected, washed with PBS, and centrifuged at 300 × g for 10 min at 4 °C. The resulting cell pellet was resuspended in PBS and used for leukocyte quantification and morphological analysis.^57^ Cell viability was assessed using the trypan blue exclusion method, and total leukocyte counts were subsequently determined using a Neubauer hemocytometer.^58^ Assessments of differential leukocyte profiles were conducted through microscopic examination of smears of cellular suspensions extracted from gastric mucosa comprised of immune cells. Leukocyte sub-classes were classified and distinguished based on their morphological characteristics like nuclear morphology and segmentation, size, ratio of cytoplasm to nucleus and cytoplasmic granules. The smears were air-dried, fixed with absolute methanol for 3 min, and stained with Wright–Giemsa solution for 3 min at room temperature. The slides were then rinsed with distilled water, air-dried, and examined under a light microscope at ×400 magnification to identify and quantify individual leukocyte populations.^59^ For biochemical tests, the mucosal layer was scraped from the stomach with a glass slide, carefully weighed, and homogenized at 10% (w/v) in cold PBS (50 mM, pH 7.4) using Teflon/glass homogenizer. The homogenate was centrifuged at 10,000 × g for 15 min at 4 °C, and the obtained supernatant was stored at −80 °C for further biochemical analyses. TBARS levels, indicating lipid peroxidation, were determined using the method of Buege and Aust.^60^ Briefly, 200 μL of the homogenized gastric mucosa sample was combined with 400 μL of 8.1% SDS, 1.5 mL of 20% acetic acid (pH 3.5), and 1.5 mL of 0.8% thiobarbituric acid and was then incubated at 95°C for 60 min. The solution was subsequently cooled, centrifuged at 10,000 × g for 10 min, and its absorption was determined at 532 nm. TBARS concentration is presented as nmol MDA/mg protein. Protein concentration was measured using the Kit (ref. 97016, Biolabo, France), where 10 μL of homogenized gastric mucosa was mixed with 200 μL of Bradford reagent, and absorbance was measured at 595 nm. MPO activity was quantified spectrophotometrically at 460 nm using 100 μL of homogenized gastric mucosa incubated with 1 mL of *o*-dianisidine dihydrochloride and hydrogen peroxide substrates; one unit is defined as degrading 1 μmol of peroxide per minute at 25°C.^61^ CRP levels were measured using a Biolabo ELISA kit (France, ref. CRP620) following the manufacturer’s instructions, using 50 μL of homogenized gastric mucosa per well. NO levels were assessed using the Griess diazotization reaction,^62^ where 100 μL of homogenized gastric mucosa was mixed with 100 μL of Griess reagent, and absorbance was read at 540 nm. H_2_O_2_ concentrations were measured by reacting 200 μL of homogenized gastric mucosa with 1.5 mL of titanium sulfate reagent, followed by absorbance measurement at 410 nm.^63^ Pepsin activity was analyzed using a stop-point assay with 500 μL of gastric juice incubated with 1 mL of 0.65% denatured hemoglobin solution at 37°C for 10 min, and absorbance was read at 280 nm.^64^ CAT activity was evaluated by measuring the decomposition of H_2_O_2_ at 240 nm using 100 μL of homogenized gastric mucosa added to 2.9 mL of 10 mM H_2_O_2_ in phosphate buffer (pH 7.0) at 25°C.^65^ SOD activity was determined using 100 μL of homogenized gastric mucosa incubated with 1.5 mL of pyrogallol solution, and inhibition of autoxidation was measured at 540 nm.^66^ GPx activity was assessed by mixing 200 μL of homogenized gastric mucosa with 800 μL of glutathione and 100 μL of cumene hydroperoxide, and measuring NADPH consumption at 340 nm.^67^ Gastric mucosal barrier integrity was evaluated by staining with 1 mL of 0.1% Alcian blue for 2 h, then washing and measuring dye absorption at 580 nm.^68^ PGE2 levels were quantified via ELISA (Bioneovan Co. China) after homogenizing 100 mg of homogenized gastric mucosa in 1 mL of ethanol containing 10 μM indomethacin. Mucosal hexosamine content was assessed by hydrolyzing extracted mucin with 4 N HCl at 100°C for 6 h, followed by reaction with 1 mL of acetylacetone and Ehrlich’s reagent, with absorbance measured at 550 nm.^69^ COX activity in homogenized gastric mucosa was determined spectrophotometrically by measuring the oxidation of N,N,N′,N′-tetramethyl-*p*-phenylenediamine at 590 nm, using 100 μL of homogenized gastric mucosa incubated with COX substrate.^70^ Gastric mucus content was assessed using the method of Corne et al.^71^ involving incubation of gastric tissue with Alcian blue and subsequent elution for spectrophotometric measurement. Gastric juice volume (GJV) analysis involved collecting gastric juice sample and then subjecting it to centrifugation at 3500 × g for 5 min at 4 °C to remove mucus and tissue particles. The volume of the clear supernatant was obtained using graduated tubes and was expressed as mL.^44^ The gastric mucosal sample was homogenized in a Tris-HCl buffer (50 mM, pH 7.4) with added sucrose (250 mM) and EDTA (1 mM). Subsequently, membrane fractions were obtained after centrifugation and ultracentrifugation (100,000 × g for 1 h). H^+^,K^+^-ATPase activity following the protocol described by Nagaya et al.^72^ using the released inorganic phosphate (Pi) measurement at 400 nm.

#### Gastric macroscopic and microscopic indices measurements

After rinsing the stomach tissue specimens with phosphate-buffered saline (PBS) to get rid of blood clots, the tissues were examined macroscopically to calculate the gastric ulcer index. Using an inverted microscope, a digital camera, ImageJ software, and a digital e-ruler for scaling, the entire area of the ulcer was measured. The total ulcer area of each stomach was calculated by summing the areas of all visible lesions and expressed as gastric ulcer area (GUA, mm^2^). The following formula was used to determine the ulcer index (UI): Ul = (Ulcer area/Total mucosa surface area) x 100. The curative index (CI) was calculated to determine the percentage of gastric mucosal protection provided by Lut treatment according to the following formula: CI (%) = [(GUA of GU group − GUA of Lut-treated group)/GUA of GU group] × 100.

For histopathological examination, the samples of the gastric mucosa were first fixed in 10% neutral buffered formalin. After fixation, the samples were then subjected to processing and embedding in paraffin wax. The sections were cut at 5 μm and stained using hematoxylin and eosin stain. Under the light microscope, alterations in histopathology such as epithelial erosion, tissue necrosis, edema, and infiltration by inflammatory cells were observed.

### Quantification and statistical analysis

Statistical analysis was performed using StatView software, Version 5.0 (SAS Institute Inc., Cary, NC, USA). Mean ± SEM is used to represent the data, and each group consists of *n* = 6 animals. One-way ANOVA was used to compare differences between groups, while Tukey’s post hoc test was performed for multiple comparisons. *p* ≤ 0.05 was taken as statistically significant. Statistical significance is represented as follows: ∗*p* ≤ 0.05. Statistical significance was determined in the following way: ∗*p* < 0.05 when compared with the normal control group; ^@^*p* < 0.05 when compared with the untreated ulcer group; ^#^*p* < 0.05 when compared with the Lut_50_-ulcer treated group; ^&^*p* < 0.05 when compared with the Lut_100_-ulcer treated group; ^α^p < 0.05 when compared with the Lut_200_-ulcer treated group; ^β^p < 0.05 when compared with Lut_200_—Opz-ulcer treated group.
